# Core Surface Flow Changes Associated With the 2017 Pacific Geomagnetic Jerk

**DOI:** 10.1029/2022GL098616

**Published:** 2022-08-05

**Authors:** K. A. Whaler, M. D. Hammer, C. C. Finlay, N. Olsen

**Affiliations:** ^1^ School of GeoSciences University of Edinburgh Edinburgh UK; ^2^ DTU Space Kongens Lyngby Denmark

## Abstract

A geomagnetic jerk was seen in *Swarm* satellite data in 2017 over the Pacific region. We invert time series of spatial gradient secular variation data between 2014 and 2020, reduced to a grid of points at satellite altitude, for spatially‐ and temporally‐regularized core surface flow. Pacific region flow acceleration was almost constant before and after the jerk, with a sharp change, especially in the azimuthal component, at the jerk epoch, despite the temporal regularization. Azimuthal acceleration is oppositely signed either side of 160°W, where it effectively vanishes, and also reverses sign at the jerk epoch. Acceleration features drift westward at about 900 km year^−1^. Unlike previous studies, the evidence presented here for low latitude waves does not depend on imposing flow equatorial symmetry, quasi‐ or tangential geostrophy, or band‐pass filtering, and has no reliance on stochastic models or numerical simulations.

## Key Points

- Inversion for core surface flow of *Swarm*‐derived secular variation spatial gradients resolves ∼50% more coefficients than vector components
- Models include cross‐equatorial flow beneath Indonesia, a notable departure from tangential geostrophy and equatorial symmetry
- Although temporal flow variability is constrained, rapid acceleration changes are required to fit data in the Pacific region around 2017

## 1 Introduction

Low Earth orbit satellite data have greatly improved the spatial coverage of the magnetic field compared to that available from ground measurements such as observations made at permanent geomagnetic observatories and repeat stations. They also enable spatial field gradients to be estimated by taking along track and, in the case of the current trio of satellites forming the European Space Agency mission *Swarm*, across track differences. These differences have greater sensitivity to the core field and its rate of change at small length scales (Kotsiaros & Olsen, 2014). Another successful innovation has been the concept of the “geomagnetic virtual observatory” (GVO), time series of field components (and their time derivatives) at a point at satellite altitude mimicking the output of a ground magnetic observatory. Satellite data in a cylindrical volume are reduced to the GVO location using a potential expansion, assuming an absence of magnetic sources. Several refinements and improvements have followed the pioneering work of Mandea and Olsen (2006), including consideration of data selection, the degree of the magnetic scalar potential expansion, the volume of the cylinder centered on the GVO point, the time interval between successive point estimates, and the inclusion of magnetic gradient observations (Hammer, Cox, et al., 2021; Hammer et al., 2022). GVO monthly mean time series have been shown to agree well with their ground counterparts, and provide global coverage, in particular, filling large gaps over regions of few permanent observatories such as the oceans (e.g., Hammer, Cox, et al., 2021).

With the abundance of high quality geomagnetic data has come the realization that there have been a number of recent instances where the time derivative of the field, or secular variation (SV), has undergone rapid change, a phenomenon known as a geomagnetic jerk (e.g., Brown et al., 2013; Courtillot et al., 1978). Geomagnetic jerks occur over a period of months. They are normally spatially localized, and often affect just one vector SV component, though some have a greater spatial reach, and may be seen in different components in different parts of the globe (e.g., De Michelis et al., 2002; Le Huy et al., 1998). Satellite data have enabled better depiction of the spatial extent and duration of jerks (e.g., Olsen & Mandea, 2007). They have been shown to originate in the core (Malin & Hodder, 1982), despite the attenuation of rapid variations by the electrically conducting mantle (e.g., Constable, 2007), and are thought to represent changes in the flow regime at the top of the Earth’s core, or an apparent change in flow associated with imperfect coupling between the mantle and core (e.g., Gubbins, 1984; Le Mouël et al., 1981). However, the difficulty in deducing rapid changes in the core surface flow from SV data and in performing direct computational simulations of the geodynamo in the appropriate numerical regime has hindered efforts to understand the details of the connection between changes in the flow and geomagnetic jerks.

**Figure 1:**
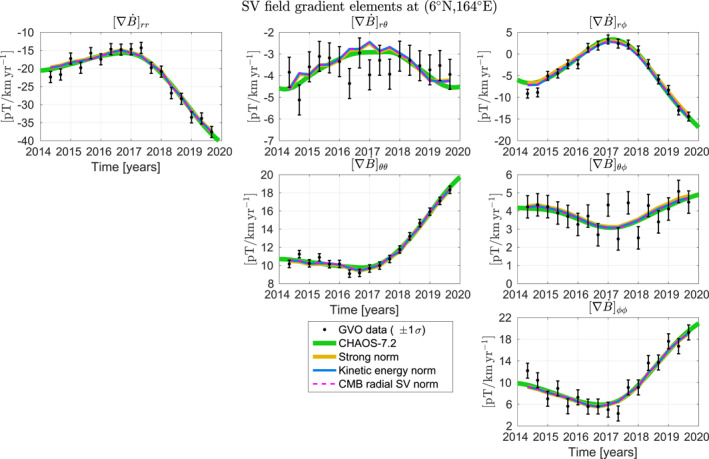
Example geomagnetic virtual observatory secular variation (SV) gradient data and model predictions at (6°N, 164°E) in the Pacific region where rapid SV change is observed at satellite altitude.

Here we produce time‐dependent core surface advective flow models by inverting GVO 4‐monthly mean time series of just the spatial gradients of the SV from *Swarm* satellite magnetic data between 2014 and 2020. Both SV vector and spatial gradient GVO time series show rapid temporal changes in the Pacific region around 2017. This jerk signal is clearest in the radial component (see Figure 5 of Hammer, Cox, et al., 2021), though visible also in the theta component (Hammer, Cox, et al., 2021, Figure 8), and is also obvious in several of the spatial gradient components (Figure 1; see also Hammer et al., 2022). We present our methods (Section 2) and data (Section 3), show our results (Section 4), and discuss them, particularly in the context of the 2017 Pacific jerk (Section 5).

## 2 Methods

The contribution of magnetic diffusion to SV is thought to be small on the timescale of years to decades (Roberts & Scott, 1965), and in common with many previous studies we assume SV results entirely from advection. The induction equation describing how the flow acts on the magnetic field to generate SV is then given by

(1) $$\dot{B}=\nabla \times \left(v\times B\right)$$

where **B** is the magnetic field and **v** the flow. Only the radial components of the field and SV are continuous across the electrical conductivity jump at the base of the mantle, so our inversion for the flow is based on the radial component of Equation 1 at the core‐mantle boundary (CMB)

(2) $${\dot{B}}_{r}+\nabla_{H}\cdot \left(vB_{r}\right)=0,$$

where ∇_*H*_ denotes the horizontal parts of the nabla operator. The solution to this equation is fundamentally non‐unique as the CMB flow **v** has two unknown components (its radial component vanishes as the CMB is a material surface) but only a single equation constrains them. A comprehensive characterization of the nature of the non‐uniqueness was given by Backus (1968), and subsequently a number of additional plausible constraints about the nature of the flow that reduce the ambiguity have been proposed (see Holme, 2015 for a summary). Our solutions assume that the flow is large‐scale and slowly varying.

We express the CMB flow in terms of its poloidal and toroidal scalars, T and S respectively, which can be expressed as truncated sums of spherical harmonics (Roberts & Scott, 1965); the coefficients in these expansions are our unknowns. The magnetic field and its SV can also be expanded in terms of spherical harmonics, and some manipulation leads to a set of linear equations relating the spherical harmonic SV coefficients to those of the flow, which can be written as

(3) $$\dot{g}=Et+Gs$$

Here, $\dot{g}$ is a vector of SV spherical harmonic coefficients $\left({\dot{g}}_{n}^{m},{\dot{h}}_{n}^{m}\right)$, and **t** and **s** are vectors of spherical harmonic coefficients $\left({t_{n}^{m}}^{c},{t_{n}^{m}}^{s}\right)$ and $\left({s_{n}^{m}}^{c},{s_{n}^{m}}^{s}\right)$ respectively of the poloidal and toroidal scalars, T and S. **E** and **G** are matrices whose values depend on the main field coefficients and either Elsasser or Gaunt integrals, respectively (e.g., Whaler, 1986). We treat the main field as known, specified by the CHAOS‐7 model (Finlay et al., 2020) to spherical harmonic degree and order 14. We invert six spatial gradient components of the SV at satellite altitude, ${\left[\nabla \dot{B}\right]}_{rr,}$ ${\left[\nabla \dot{B}\right]}_{\theta \theta ,}$ ${\left[\nabla \dot{B}\right]}_{\phi \phi ,}$ ${\left[\nabla \dot{B}\right]}_{r\theta ,}$ ${\left[\nabla \dot{B}\right]}_{r\phi }$ and ${\left[\nabla \dot{B}\right]}_{\theta \phi ,}$ where the first subscript indicates the SV component and the second the direction of the spatial derivative (although the gradient tensor is symmetric). They are related linearly to the SV spherical harmonic coefficients by (Kotsiaros & Olsen, 2014)

(4a) $$\begin{array}{lll} {\left[\nabla \dot{B}\right]}_{rr} & = & -\frac{1}{a}\sum_{n=1}^{N}\sum_{m=0}^{n}{\left(\frac{a}{r}\right)}^{n+3}\left(n+1\right)\left(n+2\right)P_{n}^{m}\left(\cos\theta \right)\\ & & \times \left({\dot{g}}_{n}^{m}\cos\left(m\phi \right)+{\dot{h}}_{n}^{m}\sin\left(m\phi \right)\right) \end{array}$$

(4b) $$\begin{array}{lll} {\left[\nabla \dot{B}\right]}_{\theta \theta } & = & \frac{1}{a}\sum_{n=1}^{N}\sum_{m=0}^{n}{\left(\frac{a}{r}\right)}^{n+3}\left(\left[{\left(n+1\right)}^{2}-\frac{m^{2}}{\sin^{2}\theta }\right]P_{n}^{m}\left(\cos\theta \right)+\cot\theta \frac{dP_{n}^{m}\left(\cos\theta \right)}{d\theta }\right)\\ & & \times \left({\dot{g}}_{n}^{m}\cos\left(m\phi \right)+{\dot{h}}_{n}^{m}\sin\left(m\phi \right)\right) \end{array}$$

(4c) $$\begin{array}{lll} {\left[\nabla \dot{B}\right]}_{\phi \phi } & = & \frac{1}{a}\sum_{n=1}^{N}\sum_{m=0}^{n}{\left(\frac{a}{r}\right)}^{n+3}\left(\left[\left(n+1\right)+\frac{m^{2}}{\sin^{2}\theta }\right]P_{n}^{m}\left(\cos\theta \right)-\cot\theta \frac{dP_{n}^{m}\left(\cos\theta \right)}{d\theta }\right)\\ & & \times \left({\dot{g}}_{n}^{m}\cos\left(m\phi \right)+{\dot{h}}_{n}^{m}\sin\left(m\phi \right)\right) \end{array}$$

(4d) $${\left[\nabla \dot{B}\right]}_{r\theta }=-\frac{1}{a}\sum_{n=1}^{N}\sum_{m=0}^{n}{\left(\frac{a}{r}\right)}^{n+3}\left(n+2\right)\frac{dP_{n}^{m}\left(\cos\theta \right)}{d\theta }\left({\dot{g}}_{n}^{m}\cos\left(m\phi \right)+{\dot{h}}_{n}^{m}\sin\left(m\phi \right)\right)$$

(4e) $${\left[\nabla \dot{B}\right]}_{r\phi }=-\frac{1}{a}\sum_{n=1}^{N}\sum_{m=0}^{n}{\left(\frac{a}{r}\right)}^{n+3}\frac{m(n+2)}{\sin\theta }P_{n}^{m}\left(\cos\theta \right)\left({\dot{g}}_{n}^{m}\sin\left(m\phi \right)-{\dot{h}}_{n}^{m}\cos\left(m\phi \right)\right)$$

(4f) $$\begin{array}{lll} {\left[\nabla \dot{B}\right]}_{\theta \phi } & = & -\frac{1}{a}\sum_{n=1}^{N}\sum_{m=0}^{n}{\left(\frac{a}{r}\right)}^{n+3}\frac{m}{\sin\theta }\left[\cot\theta P_{n}^{m}\left(\cos\theta \right)-\frac{dP_{n}^{m}\left(\cos\theta \right)}{d\theta }\right]\\ & & \times \left({\dot{g}}_{n}^{m}\sin\left(m\phi \right)-{\dot{h}}_{n}^{m}\cos\left(m\phi \right)\right) \end{array}$$

where *a* is the Earth’s mean radius, $P_{n}^{m}\left(\cos\;\theta \right)$ are Schmidt quasi‐normalized associated Legendre functions of degree *n* and order *m*. Combining all spatial gradient SV data components in Equation 4 at all GVO locations, we can write

(5) $$\dot{d}=Y\dot{g}$$

where $\dot{d}$ contains the spatial gradient data. From Equations 3 and 5 we obtain

(6) $$\dot{d}=YEt+YGs\equiv Am$$

as the set of linear equations relating the data to the flow coefficients **t** and **s**, collected in the vector **m**.

We follow the approach of Whaler et al. (2016) to find temporally smooth flows. We also apply spatial regularization, using three different norms to demonstrate robustness of our flows, particularly the changes associated with the Pacific jerk. The first, proposed by Bloxham (1988), minimizes root‐mean‐square (rms) flow second spatial derivatives (FSD) over the CMB

(7) $$\int_{\text{CMB}}\left[{\left(\nabla_{H}^{2}v_{\theta }\right)}^{2}+{\left(\nabla_{H}^{2}v_{\phi }\right)}^{2}\right]dS$$

denoted the “FSD norm.” The second minimizes the rms kinetic energy in the CMB flow

(8) $$\int_{\text{CMB}}v^{2}dS$$

(Whaler, 1986), referred to here as the “KE norm.” Finally, we minimize the rms radial SV over the core surface generated by flow advection

(9) $$\int_{\text{CMB}}{\dot{B}}_{r}^{2}dS$$

referred to here as the “SV norm.” Minimizing Equation 9 was introduced for CMB SV modeling (Gubbins, 1983, 1984), but can also be applied when inverting for flow coefficients (Whaler, 1986). The FSD and KE norms lead to diagonal spatial regularization matrices, with terms $O(n^{5})$ and O(n) respectively. The regularization matrix for the SV norm is full. For acceptable data fits, the FSD norm spherical harmonic series for T and S converge with expansions to degree 14 because of the rapid increase in regularization matrix elements with spherical harmonic degree. However, we need to extend this to degree 20 for the KE and SV norms.

Temporally and spatially regularized solutions to Equation 6, inverting data from all available epochs, are obtained via

(10) $$\hat{m}={\left(A^{T}{C_{e}}^{-1}A+\lambda_{v}{C_{m}}^{-1}+\lambda_{t}\;D^{T}D\right)}^{-1}A^{T}{C_{e}}^{-1}\dot{d}$$

where $\hat{m}$ is our estimate of **m**, containing the unknown flow coefficients at every epoch. **C**_*e*_ consists of 6 × 6 data covariance matrices for each GVO location with the variance of each gradient datum arranged along the diagonal, and zeroes elsewhere. **C**_*m*_ is the *a priori* model covariance matrix spatially regularizing the flow, whose elements depend on the choice of norm, and **D** is the first difference penalty matrix restricting changes in the flow between epochs (see Whaler et al. (2016)). *λ*_*v*_ and *λ*_*t*_ are damping parameters controlling how much spatial complexity and temporal variability are allowed. For the KE and SV norms, we apply different amounts of spatial damping to the toroidal and poloidal parts of the flow, using damping parameters $\lambda_{v_{T}}$ and $\lambda_{v_{P}}$.

## 3 Data

We first summarize briefly the method for obtaining the *Swarm* GVO time series representing the core field, described in detail by Hammer, Cox, et al. (2021), and then outline the differences to produce spatial gradient GVO time series. Finally we describe how SV GVO estimates are formed.

The algorithms use both along‐track measurement means and differences along a single satellite orbit, and across‐track measurement means and differences from the two lower orbit *Swarm* satellites Alpha and Charlie that fly effectively side‐by‐side. Satellite measurements, provided in an Earth‐centered spherical coordinate system, are selected based on a set of criteria that should ensure minimal contribution from ionospheric and magnetospheric fields (and their induced signals). Estimates of the magnetospheric, ionospheric, lithospheric and main fields are removed to leave measurement “residuals.” Field estimates are derived at specific target positions, referred to as GVO locations, based on such residuals collected within a cylinder of radius 700 km and over a period of 4 months; this ensures reasonable coverage within the cylinder and mitigates local time biases. Field residuals are rotated from the Earth‐centered spherical system to a right‐handed local topocentric Cartesian system centered on the GVO location, and are then fitted to a magnetic scalar potential expanded to third order. GVO residual field components at the GVO location and central time of the 4‐month window are then calculated from the potential and transformed back to the Earth‐centered spherical coordinate system. In a final step, field estimates at the GVO location and central time, from the same time‐dependent main field model as was initially removed, are added back to give the GVO vector field data product, comprised of the three orthogonal field components *B*_*θ*_, *B*_*ϕ*_ and *B*_*r*_ at each GVO location. Uncertainties, assumed time‐independent and spatially uncorrelated, are calculated independently for each field component at each GVO location. 300 GVO locations at a height of 490 km are distributed on an approximately equal area grid, with a sampling radius of 700 km, giving no overlap and almost no gaps (see Figure 3 of Hammer, Cox, et al., 2021).

As described by Hammer et al. (2022), essentially the same processing chain is used to derive GVO time series for the field gradients. The satellite data residuals are once again modeled by a cubic expansion of the potential in Cartesian coordinates, but this time the residual gradient components rather than the vector components at the GVO location are estimated. We note that spatial gradients are less sensitive than vector components to unmodeled (or inaccurately modeled) magnetospheric fields because they are large‐scale (Hammer et al., 2022; Kotsiaros & Olsen, 2014). In the final step, the main field model prediction for the gradient components at the GVO location and time is added back, again using the same time‐dependent model as was initially subtracted from the measurements. As for the vector field, data uncertainties are estimated assuming time‐independence and no spatial correlation.

Estimates of the SV (vector components or their spatial gradients) are calculated using annual first differences, ascribed to the central time between the two values whose differences are taken. Examples of GVO SV gradient time series with their one standard deviation error bars for a location in the central Pacific are shown in Figure 1. We invert GVO SV gradient time series consisting of 17 estimates at 4‐monthly intervals covering the period from 2014.33 to 2019.67.

## 4 Results

Although Equation 10 is amenable to direct inversion with just 17 epochs of data, we took advantage of the sparseness of the system and used the conjugate gradient algorithm with Jacobi preconditioning. We set *λ*_*t*_ to 1,000, which was found by Whaler et al. (2016) to be a good compromise between minimizing acceleration and allowing the flow to evolve where necessary to fit the data. Our preferred spatial damping parameters give an overall rms misfit, normalized by the data uncertainties, of 0.93—lighter damping led to unstable, unconverged solutions, whereas heavier damping reduced the amplitude of the flow, but not its pattern, and the data fit. This misfit to the gradient GVO data is similar to that obtained by CHAOS‐7. To prevent small‐scale (represented by spherical harmonic degrees 17–20) poloidal flow components from having large amplitude in the KE norm case, we applied heavier damping to the poloidal part (Table S1 in Supporting Information S1). Increasing the poloidal damping parameter by a factor of 100 or more compared to the toroidal damping parameter does not affect the overall misfit, so we regard these small‐scale poloidal components as unresolved. For the SV norm, the toroidal part of the flow is slightly more heavily damped (Table S1 in Supporting Information S1) to improve convergence. Predictions by the flows and CHAOS‐7 are superimposed on an example GVO SV gradient time series in Figure 1. The flows reproduce the observed rapid changes in SV gradients associated with the 2017 jerk, and show no obvious bias.

**Figure 2:**
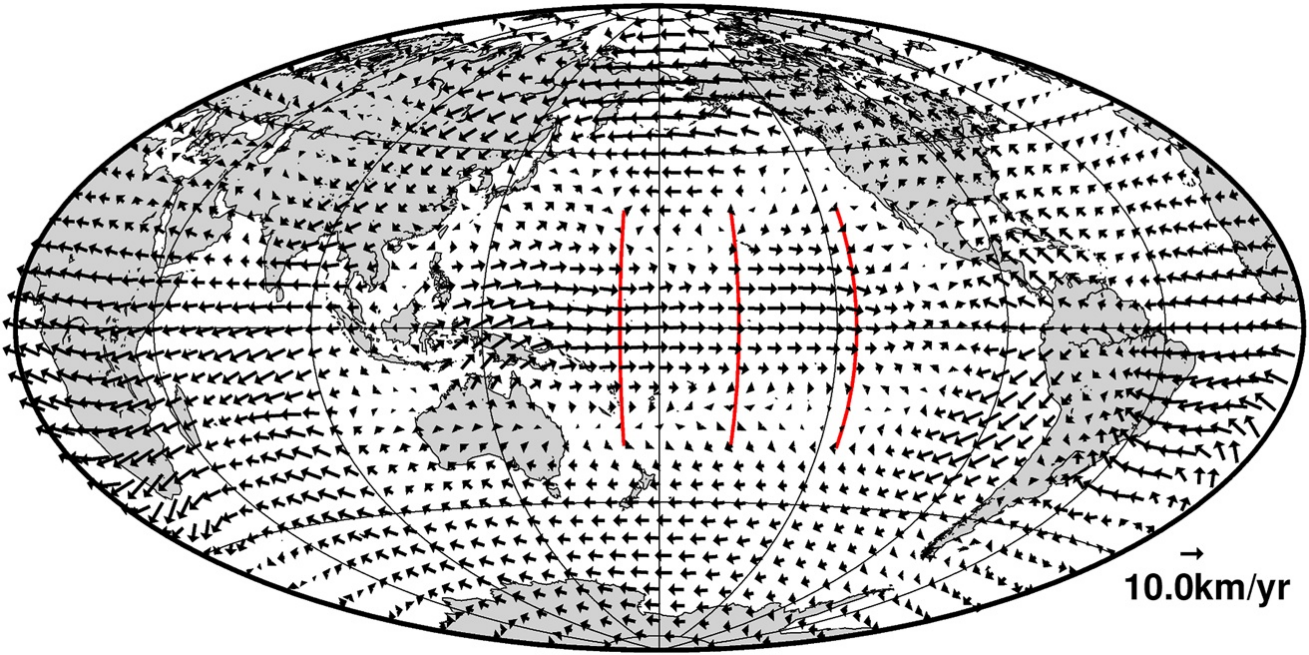
Average flow second spatial derivatives norm core‐mantle boundary flow obtained with the parameters of Table S1 in Supporting Information S1. The locations of the azimuthal acceleration plots in Figure 3 are indicated by the red lines. Coastlines are shown for reference.

Global flow plots (e.g., Figure 2), although different in detail for the different spatial normalizations, retain features familiar from inversions of SV vector component data and spherical harmonic models—westward drift at the core surface in an equatorial band centered on the Greenwich meridian; a band of eastward flow along the equator beneath the Pacific (Gillet et al., 2019), and somewhat slower flows elsewhere; the planetary‐scale eccentric gyre (Pais & Jault, 2008) is also evident. In Figure 2 the gyre is rather diffuse because of the strong spatial regularization; for the other spatial normalizations (see Figure S1 in Supporting Information S1), it is better localized but with other small‐scale features superimposed.

We resolved approximately 160 flow coefficients per epoch in all cases (as estimated by the trace of the resolution matrix), compared to ∼100 when inverting vector SV components from either observatories (Whaler et al., 2016) or GVOs (Table S1 in Supporting Information S1). We divide the trace into its toroidal and poloidal parts in Table S1 in Supporting Information S1, showing that flows derived with the KE norm resolve the toroidal flow better (because of the heavier damping of the poloidal part) while those obtained with the FSD and SV norms resolve more poloidal coefficients.

**Figure 3:**
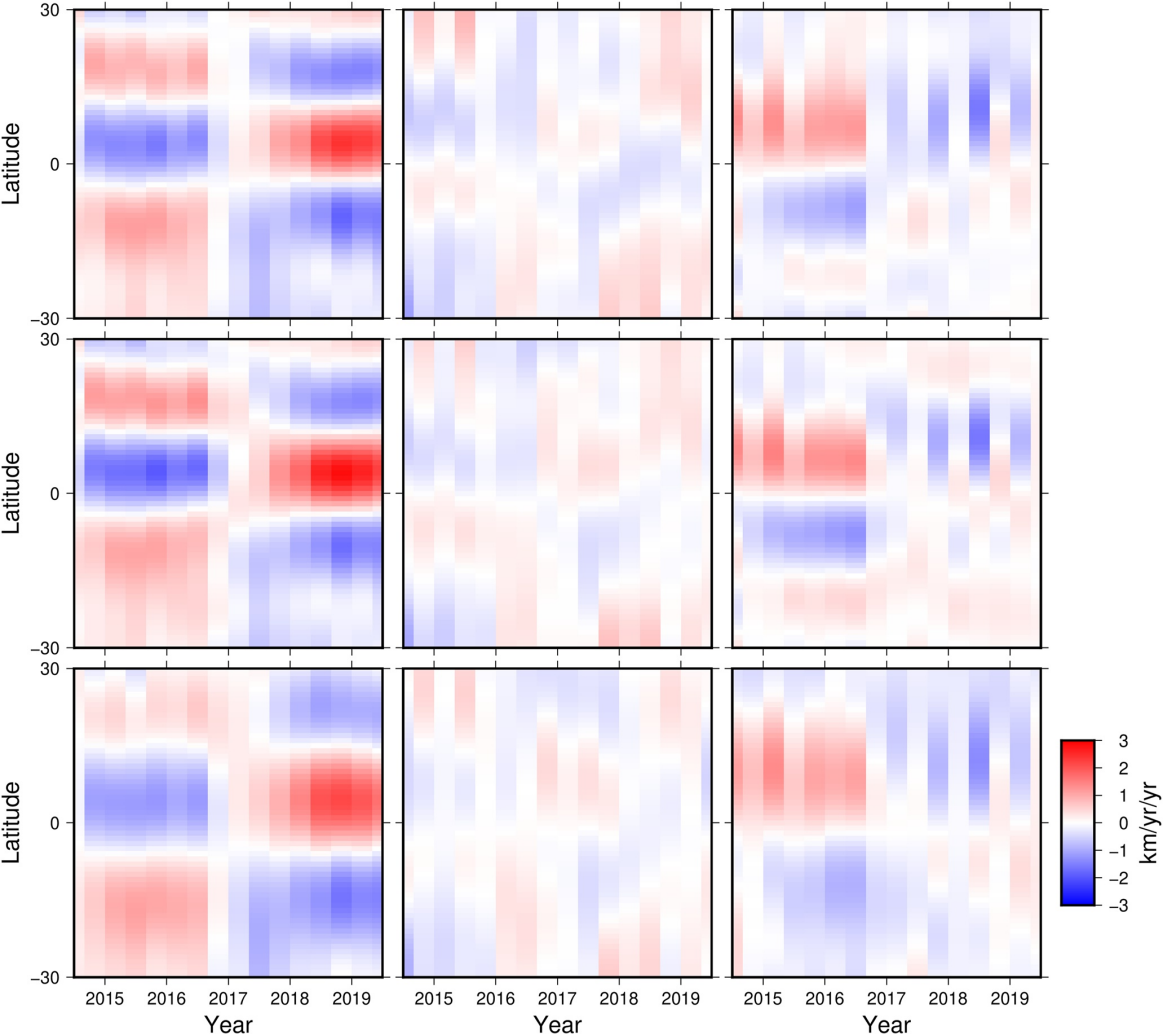
Time evolution of the azimuthal acceleration component at three longitudes (separated by 30°) beneath the Pacific, plotted as a function of latitude (°), for flows with different spatial norms. Left to right are 170°E, 160°W, and 130°W. Top to bottom are for the core radial secular variation, kinetic energy and flow second spatial derivatives norms.

Viewed globally, it is hard to discern any change in flow over the time interval studied. However, by focusing in on a smaller region, beneath the Pacific where a jerk occurred in 2017, we find distinct and rapid changes in all three flows at that time. Since the flows are temporally regularized, these rapid changes must be required to fit the data. The general consistency in the obtained accelerations, despite the different spatial regularizations, points toward a strong association with the jerk. The change is sharply defined at the end of 2016, predominantly to the azimuthal flow component, where azimuthal acceleration reverses sign; it is also of opposite sign beneath the eastern and western Pacific, with little change at a longitude of 160°W. These sudden sign changes in azimuthal acceleration occur simultaneously at all low latitudes. Figure 3 illustrates the azimuthal flow accelerations at the three Pacific longitudes shown on Figure 2. Accelerations were calculated as first differences of the flow, assigned to the central time point, with no smoothing. At 130°W, the accelerations are smaller after the jerk and the reversal of the pattern is less well resolved, especially south of the equator. The corresponding CMB radial secular acceleration change (between 2014 to 2017 and 2017 to 2020) in the Pacific region has two peaks of opposite sign (Finlay et al., 2020), the positive one near 170°E, where the azimuthal flow acceleration is largest both before and after the jerk, the negative one around 160°W, where the azimuthal flow acceleration is very small at all epochs. At 130°W, where the azimuthal flow acceleration has the opposite pattern to that at 170°E, there is essentially no change in CMB radial secular acceleration.

**Figure 4:**
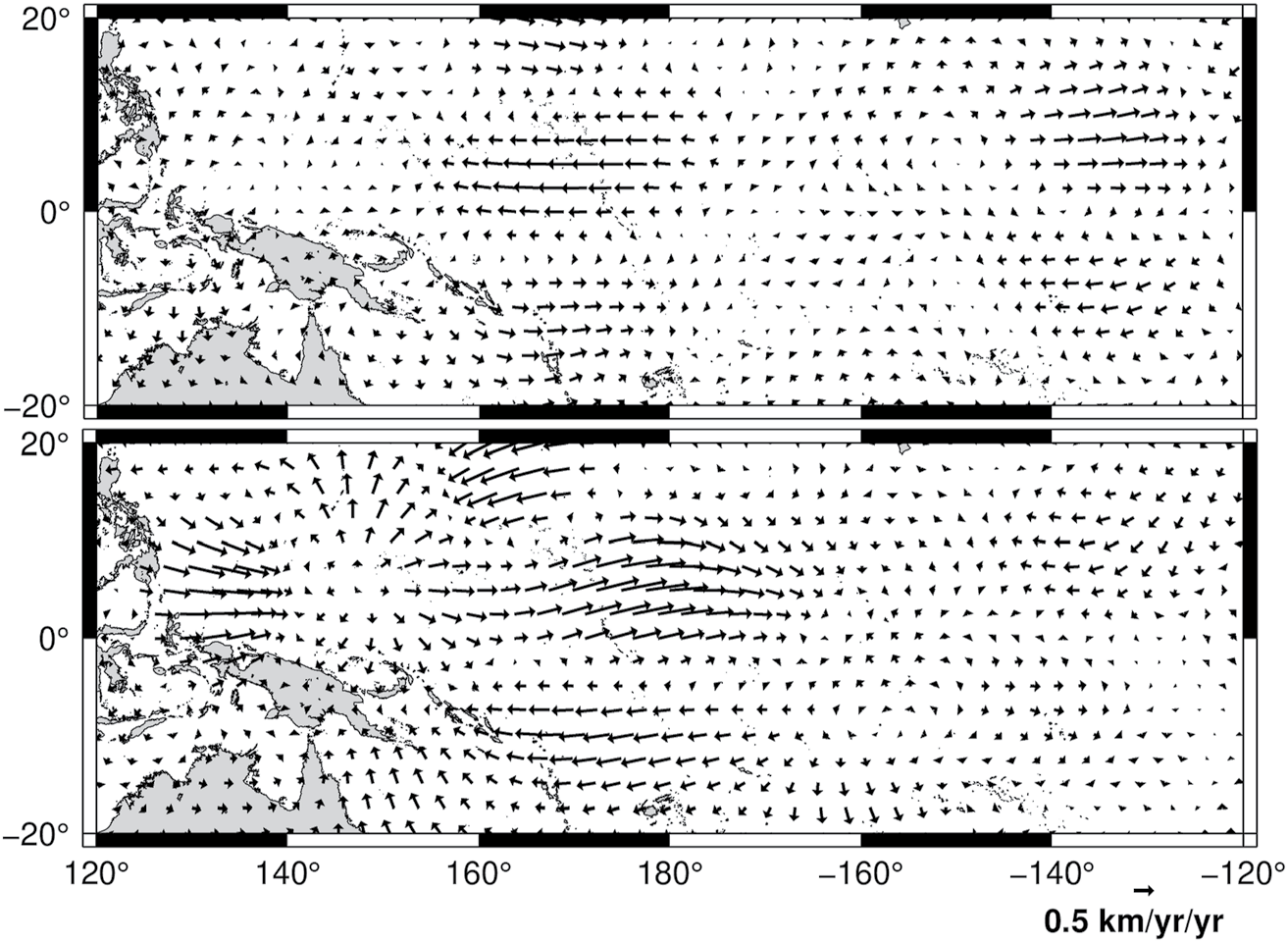
Average acceleration over the central Pacific region before (top) and after (bottom) the jerk for the core radial secular variation norm flow (for the other two norms, in Figure S2 in Supporting Information S1). Their division into various components is given in Table S2 in Supporting Information S1.

Based on our observation that the azimuthal acceleration at either side of the jerk epoch is almost steady (Figure 3), we calculated the average acceleration before and after the jerk, shown over the Pacific region in Figure 4 for the SV norm, and in the Supporting Information S1 for the other two spatial norms. The acceleration is higher in the Pacific region, locally reaching of order 1 km year^−2^ compared to global rms values of 0.32–0.42 km year^−2^ (Table S2 in Supporting Information S1). Pacific accelerations are higher after the jerk than prior to it, whereas the globally‐averaged values are similar, regardless of the spatial norm (Table S2 in Supporting Information S1). Standard deviations from time‐averaging the acceleration coefficients before and after the jerk are very small, indicating that individual values differ little from their means, and hence that accelerations globally are essentially constant before and after the jerk. Propagating these standard deviations into uncertainties on point accelerations gives values in the range 0.02–0.03 km year^−2^ before and 0.02–0.04 km year^−2^ after the jerk over the Pacific area (less than the size of the arrowheads on Figure 4), demonstrating that the change in acceleration associated with the jerk is well resolved. In the Pacific region, the acceleration is dominated by its azimuthal component but has more energy in its tangentially ageostrophic and equatorially asymmetric components than the flow itself (Table S2 in Supporting Information S1).

Features of the azimuthal flow acceleration shown in Figures 3 and 4 can be traced moving westwards across the Pacific from 2014 to 2020 in all three flow models, most noticeably near latitude 10°N as seen in time‐longitude plots in Figure S3 in Supporting Information S1. For example, a region of eastwards flow acceleration can be followed from 130°W in 2015 to 170°E in 2019 (diminishing in amplitude around the time of the jerk in 2017 when it is near to 160°W), with a corresponding drift speed of approximately 900 km year^−1^ at the core surface. Features with the opposite sign (i.e., foci of westward acceleration) are also seen to drift westwards. It is however difficult to be precise about the drift speed given the length‐scale of the features and the short time interval. Details also depend somewhat on the maximum degree of the acceleration; we implemented a truncation at degree 14.

## 5 Discussion

The flows derived here from spatial gradient GVO SV data are not constrained to be tangentially geostrophic and equatorially symmetric, but their global energy is predominantly in these components (Table S2 in Supporting Information S1). However, non‐tangentially geostrophic and equatorially anti‐symmetric flow features are locally important during the *Swarm* satellite era. In particular, there is (northwards) cross‐equatorial flow beneath Indonesia that is neither tangentially geostrophic nor equatorially symmetric, as previously noted in flow models for 1975–1980 by Bloxham (1989). In addition, the azimuthal flow component beneath most of the Pacific is eastward, with speeds of up to ∼10 km year^−1^ (e.g., Figure 2), although the solid body rotation coefficient $t_{1}^{0}$ is negative (∼−6.0, ∼−6.1, and ∼−4.7 km year^−1^ for the SV, FSD, and KE norm models respectively), consistent with westward drift.

Flow accelerations also show significant departures from tangential geostrophy and equatorial symmetry. Beneath the Pacific, the ageostrophic and tangentially geostrophic components contribute roughly equally to accelerations at 170°E. All are small at 160°W, but the acceleration at 130°W is predominantly tangentially geostrophic and equatorially anti‐symmetric in the models presented here. In the Pacific region north of the equator, the symmetric and anti‐symmetric accelerations sum constructively, whereas there is considerable cancellation south of the equator.

The alternating sign of the low latitude azimuthal flow acceleration at 170°E and 130°W, and the rapid westward drift of associated flow acceleration features found in our flows, adds to an emerging picture of rich core flow dynamics at low latitudes. Using ground observatory data and an earlier version of vector component GVO data, Kloss and Finlay (2019) also found sign changes in low latitude azimuthal flow acceleration, but they adopted a more restricted time‐dependent flow model based on quasi‐geostrophic inertial modes. In the time‐interval 2014–2018 where our model overlaps theirs we find some similar features, for example, the westward acceleration at 170°E that changes sign to eastwards by 2018. However our flows are less equatorially‐symmetric, with the largest accelerations in the equatorial belt north of the equator (see Figures 3 and 4), and have stronger cross‐equatorial flow, notably under Indonesia.

Previous studies have provided evidence for low latitude westward propagating oscillations at speeds similar to those suggested here. Chulliat et al. (2015) performed power spectral analysis in the latitude range ±15° of a secular acceleration pulse in 2012 that preceded a geomagnetic jerk. Energy in the secular acceleration of the magnetic field was found to be predominantly in the equatorially symmetric component, concentrated at azimuthal wave number −6 (i.e., westward, with angular wavelength 60°), propagating at a speed of ∼550 km year^−1^ with a period of ∼8 years. They suggested that such signals may be compatible with equatorial magneto‐Coriolis waves propagating in a stably stratified layer adjacent to the CMB. Gillet et al. (2022) presented indications of westward propagating flow oscillations at low latitudes by filtering their stochastic model of core flow (that was derived from the CHAOS‐7 field model) to retain only periods between 4 and 9.5 years. They interpret these oscillations as quasi‐geostrophic magneto‐Coriolis modes and find that the oscillations have a strong signature in the azimuthal flow component, an amplitude of 3 km year^−1^, period about 7 years, travel westwards at about 1,500 km year^−1^, and are particularly distinct after 2012 under the Pacific. Our flows show a sign change in the azimuthal flow acceleration taking place within 1 year at 170°E and 130°W, with the acceleration features appearing to move rapidly westwards between these locations. The azimuthal flow acceleration patterns we find are less equatorially symmetric than the flow patterns isolated by Gillet et al. (2022) using bandpass filtering between 4.5 and 9 years, for example, at 170°E, and less strongly confined to the equator, as we find significant flow accelerations at latitudes 15°N–30°N. Power spectral analysis of our models is challenging since we have relatively few time samples, so results should be treated with caution, but Figure S4 in Supporting Information S1 presents frequency‐azimuthal wave number power spectral density plots derived from time‐longitude plots of the azimuthal flow acceleration at 10°N for each of the three spatial norms. These were derived by subtracting the temporal mean at each longitude, padding with zeros, carrying out a 2D fast Fourier transform (in time and longitude) and then calculating the power of the resulting Fourier modes. For more details on frequency‐wave number analysis see Wheeler and Kiladis (1999). Similar analysis has previously been applied in geomagnetism by Finlay and Jackson (2003) and Chulliat et al. (2015). Results consistently show a dominant wave number −2 signal, but with peaks at other wave numbers, most noticeably −6 and +3, with periods of 6–8 years. Our result is obtained simply from direct inversion of SV data, without reliance on stochastic or numerical dynamo models, and we have not performed period filtering or imposed geometrical constraints such as tangential geostrophy, quasi‐geostrophy or equatorial symmetry. Hence a variety of different approaches support the existence of rapidly westward propagating equatorial waves, possibly magneto‐Coriolis modes, at the top of the core.

It has been suggested that sign changes in the non‐zonal azimuthal flow acceleration can cause geomagnetic jerks (Kloss & Finlay, 2019), for example, via hydromagnetic waves arriving at low latitudes at the core surface (Aubert & Finlay, 2019). Our results, derived by a rather different technique (that in particular allows considerable equatorial asymmetry), also show that sign changes of the azimuthal flow acceleration are associated with the 2017 Pacific jerk, as well as possible indications of rapid westward drift of the associated flow acceleration patterns. This suggests that such features be investigated further as candidates for generating jerk signatures.

We find that GVO SV gradient data are especially suitable for core flow investigations because they are less sensitive to contamination by magnetospheric fields and resolve more flow coefficients, with deeper insights expected from a long *Swarm* satellite mission. It may be possible to increase the resolution further if new satellites can provide additional local time coverage; this would help improve knowledge of magnetospheric, ionospheric and related induced field components that limit present studies.

## Supporting information

Supporting Information S1

## Data Availability

The GVO gradient tensor data are available from https://doi.org/10.11583/DTU.14695590.v2, (Hammer, Finlay, & Olsen, 2021).
