# Supplementary material for: Core Surface Flow Changes Associated With the 2017 Pacific Geomagnetic Jerk
Source: Geophys Res Lett. 2022 Aug 5;49(15):e2022GL098616. doi: 10.1029/2022GL098616 (PMC9539959; doi:10.1029/2022GL098616)
Supplement: Supplementary file 1 — Supporting Information S1 [file GRL-49-e2022GL098616-s001.pdf]

AGU PUBLICATIONS

Geophysical Research Letters

Supporting Information for

Core surface flow changes associated with the 2017 Pacific geomagnetic jerk

K A Whaler[1], M D Hammer[2], C C Finlay[2], N Olsen[2]

[1]School of GeoSciences, University of Edinburgh, James Hutton Road, Edinburgh EH9 3FE, UK

[2]DTU Space, Electroveg Bygning 327, 2800 Kongens Lyngby, Denmark

Contents of this file

Figures S1 to S3
Tables S1 and S2

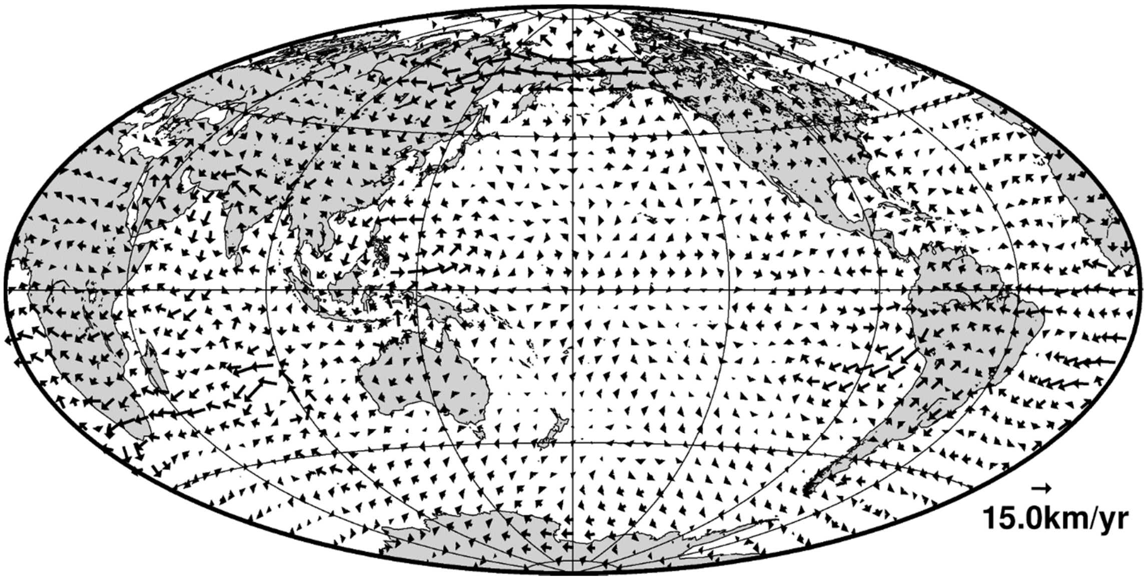

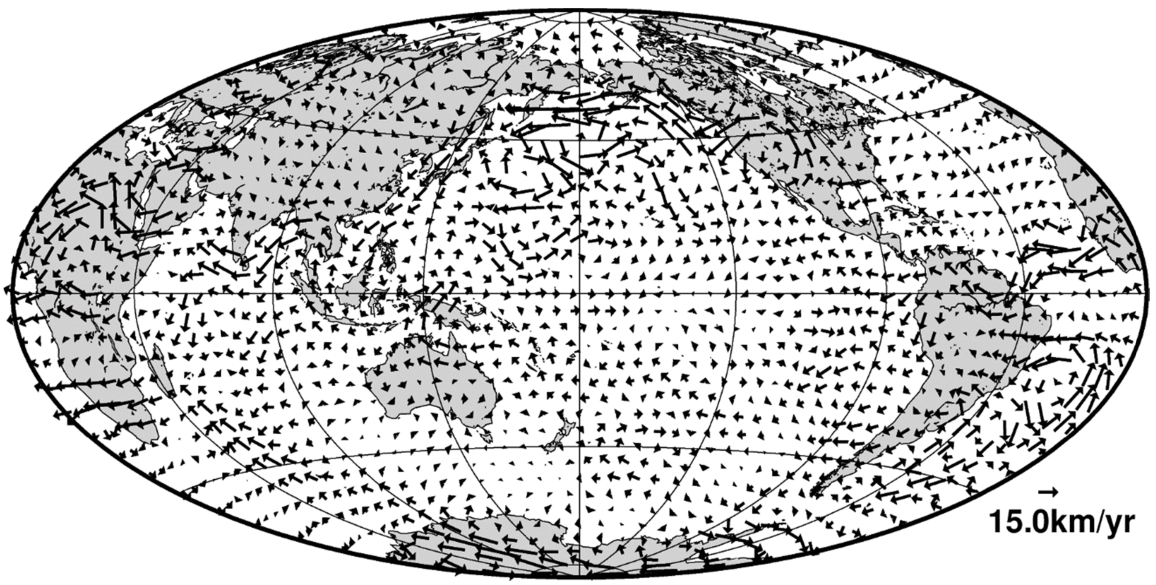

Figure S1. As for Figure 1 in the main text but for KE (top) and SV (bottom) norms. Note reference vector is 15 km yr^{-1} here, compared to 10 km yr^{-1} in the main text.

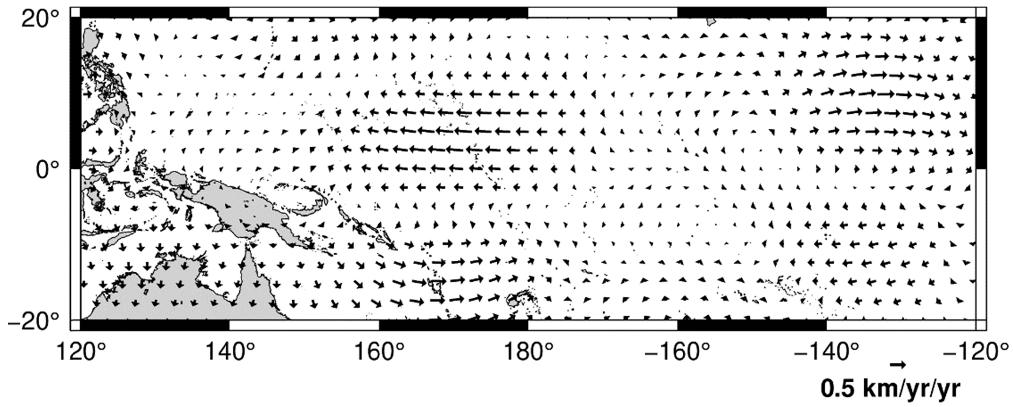

a)

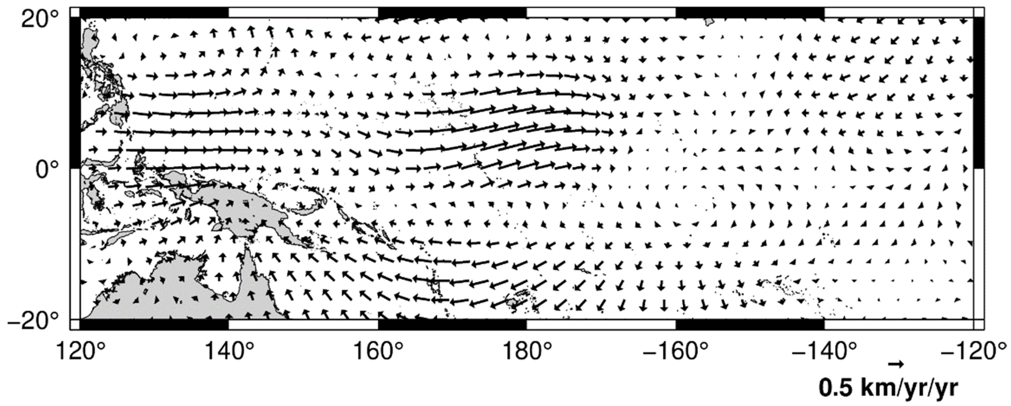

b)

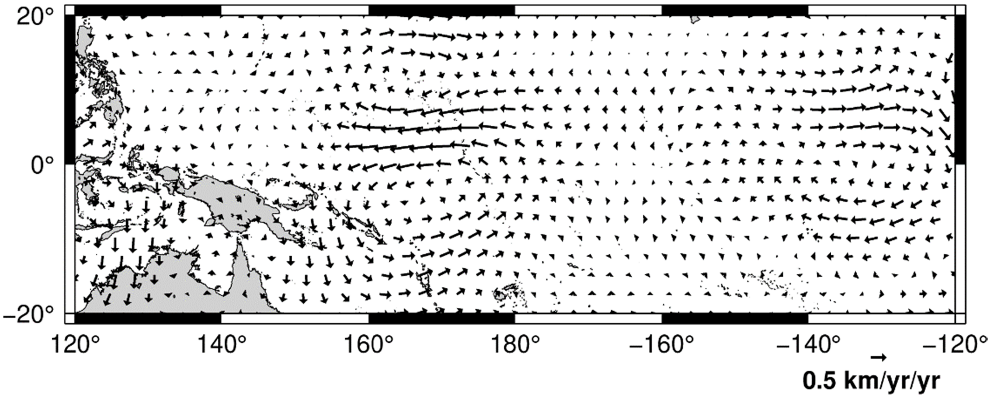

c)

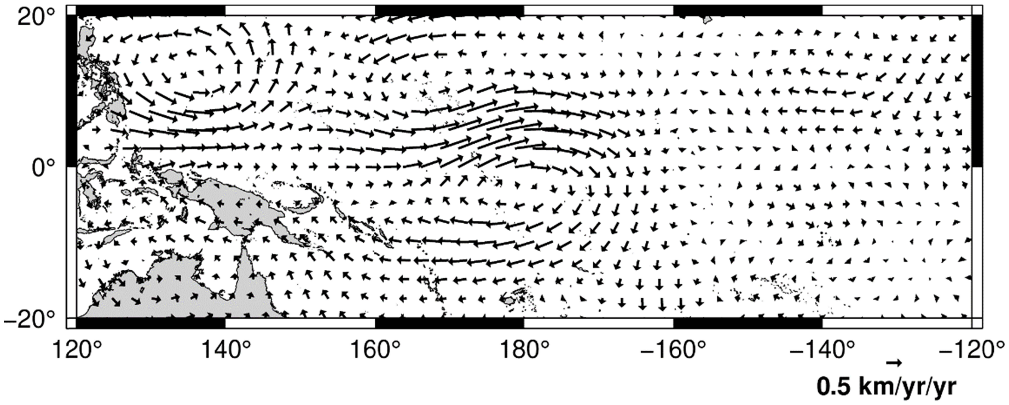

d)

Figure S2. Average acceleration before and after the 2017 jerk for the other two spatial norms. a) FSD norm before the jerk; b) FSD norm after the jerk; c) KE norm before the jerk; d) KE norm after the jerk.

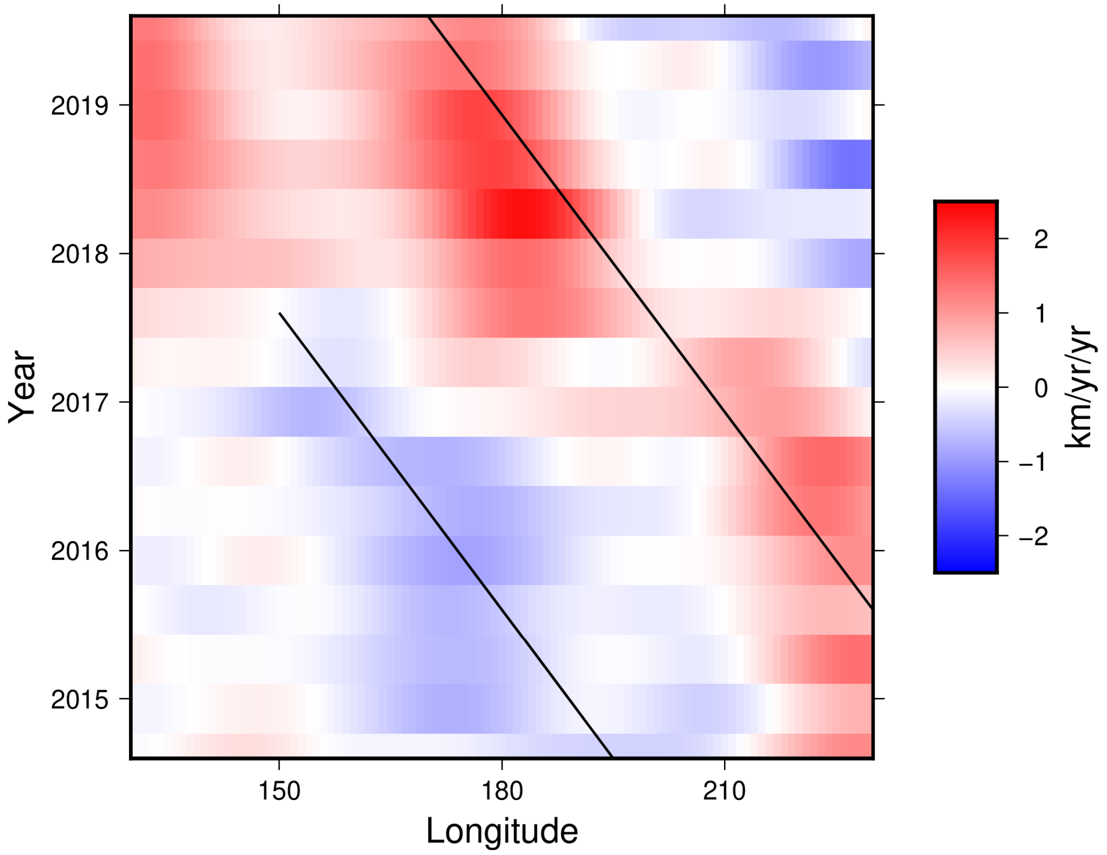

Year
2019
2018
2017
2016
2015
150
180
210
Longitude
km/yr/yr
2
1
0
−1
−2

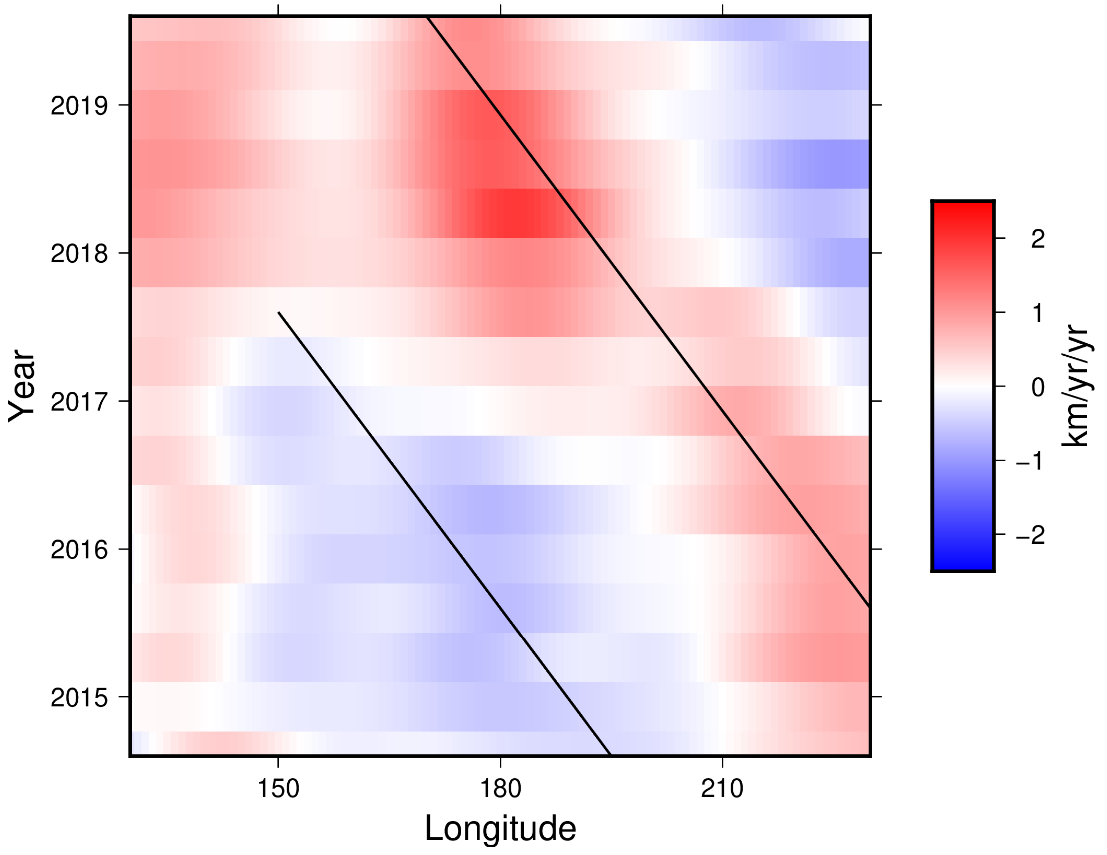

Year
2019
2018
2017
2016
2015
150
180
210
Longitude
km/yr/yr
2
1
0
−1
−2

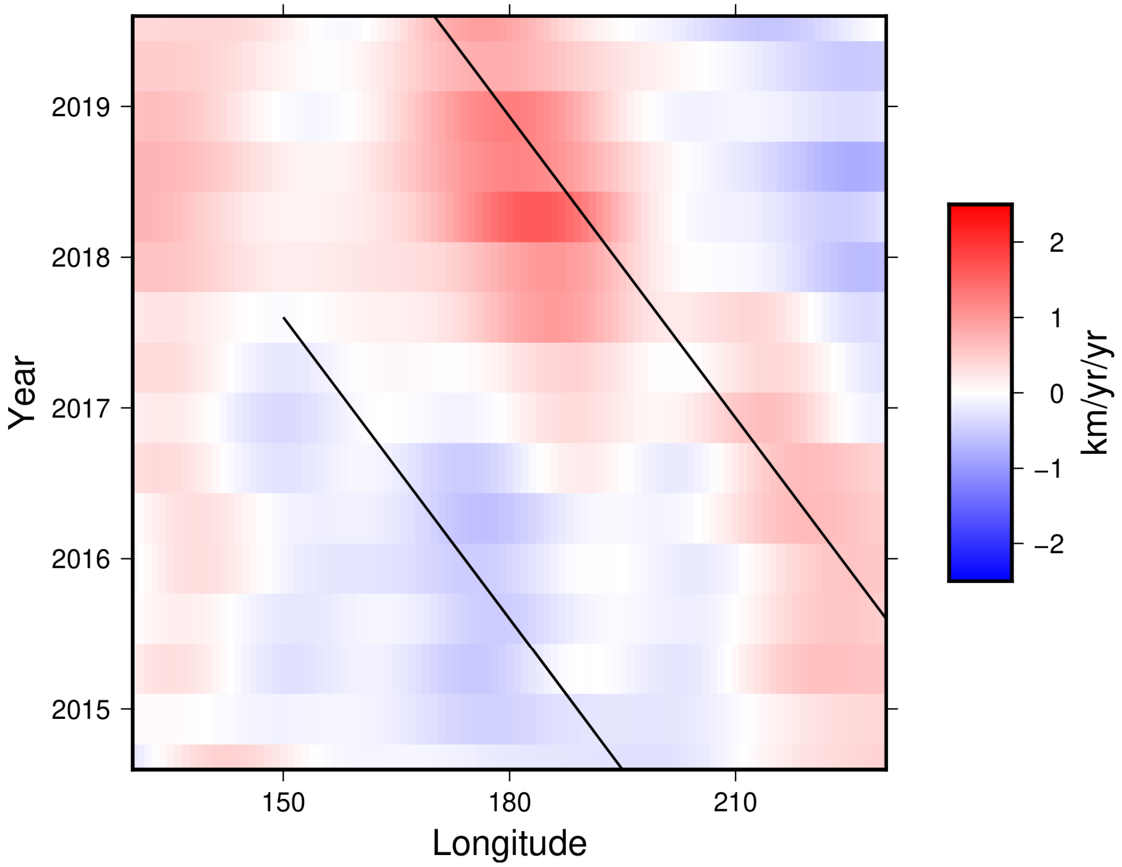

Figure S3. Azimuthal acceleration as a function of time and longitude at 10°N for FSD (top), KE (middle) and SV (bottom) norms. Acceleration for the KE and SV norms is truncated to degree 14. Superimposed lines follow, by eye, the maxima and minima; they are the same on all plots and both lines on each plot have the same slope, corresponding to a speed of ~900 km yr^{-1}.

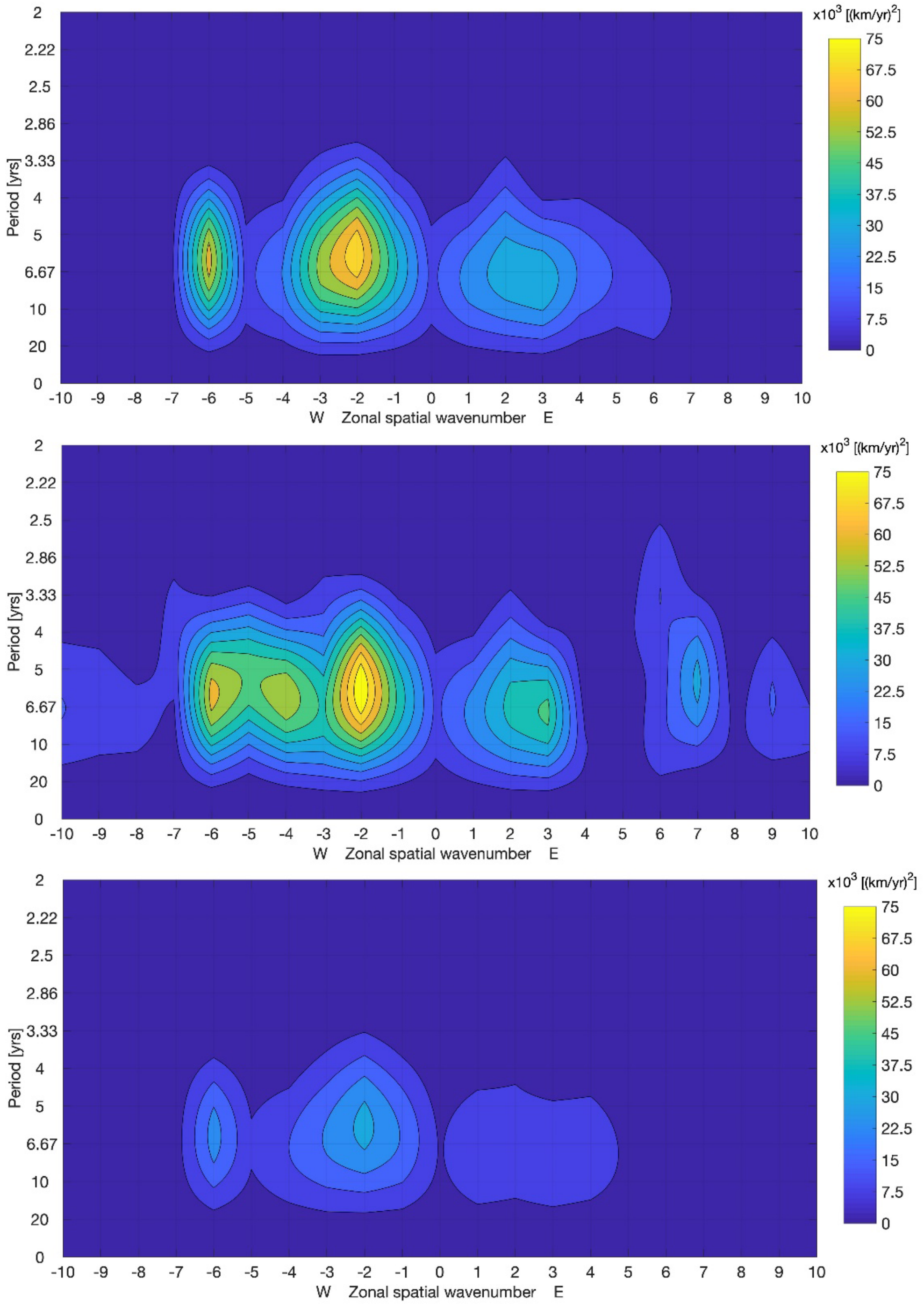

Figure S4. Power spectral density plots of the azimuthal acceleration after detrending by removing the temporal mean, at 10°N latitude. Results at other low latitudes are similar. The exact periods of the peaks depend on the detrending method employed, and they are comparable with the length of the time series, so should be interpreted with caution. Top FSD, middle KE and bottom SV norms.

Spatial norm	λ		Tr		$\overline{v}$ (km yr^{-1})						
	v_T	v_P	T	P	Total	T	P	G	AG	S	AS
FSD	10^{-4}	10^{-4}	52	103	12.6	11.6	4.8	11.3	5.4	11.7	4.6
KE	0.1	10	146	16	12.4	12.4	0.5	11.7	3.9	9.7	7.7
SV	$2\ 10^{-6}$	$8\ 10^{-7}$	44	120	19.2	18.5	5.2	17.7	7.5	14.8	12.3
FSD	$5\ 10^{-4}$	$5\ 10^{-4}$	35	67	11.9	11.0	4.5	10.6	5.5	10.7	5.2

Table S1. Numbers characterizing the flows obtained from the GVO SV data over 17 4-month epochs in the period 2014-2020.The first three rows summarize results from gradient data, and the final line is for vector data. λ_t was 1000 and the normalized misfit is 0.93 for all inversions. Tr is the effective number of toroidal (T) and poloidal (P) flow coefficients, and the rms speeds $\overline{v}$ are given for the total, torodial, poloidal, tangentially geostrophic (G), ageostrophic (AG), equatorially symmetric (S) and asymmetric (AS) parts. They are shown for a single epoch but vary very little over the period studied.

	Total	T	P	G	AG	S	AS
KE Global							
Pre-jerk	0.42	0.41	0.09	0.37	0.21	0.30	0.30
Post-jerk	0.37	0.36	0.09	0.32	0.18	0.24	0.28
FSD Global							
Pre-jerk	0.32	0.22	0.23	0.19	0.25	0.23	0.22
Post-jerk	0.32	0.25	0.20	0.24	0.22	0.24	0.21
SV Global							
Pre-jerk	0.34	0.24	0.24	0.22	0.25	0.24	0.24
Post-jerk	0.34	0.25	0.22	0.24	0.24	0.23	0.24
KE Pacific							
Pre-jerk	0.56	0.55	0.09	0.46	0.29	0.36	0.42
Post-jerk	0.79	0.63	0.14	0.70	0.36	0.47	0.63
FSD Pacific							
Pre-jerk	0.43	0.36	0.25	0.30	0.28	0.28	0.33
Post-jerk	0.70	0.59	0.36	0.55	0.40	0.51	0.47
SV Pacific							
Pre-jerk	0.45	0.35	0.28	0.32	0.30	0.28	0.35
Post-jerk	0.71	0.55	0.44	0.53	0.45	0.47	0.53

Table S2. rms accelerations globally and over the Pacific region before and after the jerk for the three spatial norms. Values over the Pacific region are calculated on a 1°x1° grid covering the area of Figure 4. Labels of parts as in Table S1. All values in km yr^{-2}.
